# Peanut Meal as a Sustainable Alternative to Soybean Meal in Laying Hen Diets

**DOI:** 10.3390/ani16101541

**Published:** 2026-05-18

**Authors:** Isidro Argentina Chemane, Gabriel Henrique Nacamura da Silva, Michele Bernardino de Lima, Erikson Kadoshe Raimundo, Rita Brito Vieira, Larissa Oliveira dos Santos, Deisy Carolina Celis Alba, Manoel Garcia Neto, Edney Pereira da Silva

**Affiliations:** 1 Department of Animal Sciences, School of Agricultural and Veterinary Sciences, São Paulo State University—UNESP, Jaboticabal 14884-900, SP, Brazil; 2 Department of Animal Production and Health, School of Veterinary Medicine, São Paulo State University—UNESP, Araçatuba 16050-680, SP, Brazil; michele.bernardino@unesp.br (M.B.d.L.); m.garcia@unesp.br (M.G.N.)

**Keywords:** alternative feed ingredients, environmental impact, feed cost

## Abstract

This research evaluated levels of peanut meal inclusion in diets for laying hens. A total of 200 Hisex White hens, 72 weeks of age, were used in a completely randomized design with five treatments and ten replicates of four hens each. Treatments consisted of replacing soybean meal with peanut meal at 0, 25, 50, 75, and 100%. Over a 70-day period, feed intake, egg production, egg weight, egg mass, feed conversion, and internal and external egg quality were evaluated. Economic analyses of diets and estimates of the carbon footprint were also conducted based on life cycle assessment data of the ingredients. Total replacement of soybean meal with peanut meal did not significantly affect productive performance or egg quality (*p* > 0.05). Increasing levels of peanut meal inclusion linearly reduced feed cost, providing savings of up to US$ 41.81 per ton. In addition, a progressive reduction in the carbon footprint of the diets was observed, reaching a decrease of 26.37% in CO_2_ equivalent emissions. Peanut meal can fully replace soybean meal in laying hen diets without compromising productive performance or egg quality while reducing feed costs and environmental impact.

## Simple Summary

Peanut meal, a by-product produced after oil is extracted from peanuts, may be a promising alternative protein source. The objective of this study was to evaluate the use of peanut meal as a replacement for soybean meal in diets for laying hens and to assess its effects on bird performance, egg quality, feed cost, and environmental impact. Hens were fed diets in which soybean meal was progressively replaced with peanut meal. The study evaluated feed intake, egg production, egg weight and quality, feed cost, and environmental impact based on greenhouse gas emissions associated with feed production. The results showed that replacing soybean meal with peanut meal did not reduce egg production or egg quality. Feed costs decreased, and the environmental impact of the diets was also reduced, indicating that peanut meal can be a practical and sustainable protein source for laying hens.

## 1. Introduction

In the poultry industry, approximately 70% of production costs are associated with bird nutrition [1,2,3,4]. This scenario reinforces the continuous need for nutritional strategies capable of reducing production costs, including the use of alternative ingredients in diet formulations [5,6,7]. Soybean meal 46% has been, for decades, the main protein source used in diets for laying hens. This ingredient presents an adequate chemical and energy composition for poultry feeding, particularly due to its favorable amino acid profile and high digestibility coefficient [8,9]. However, its use has limitations associated with price volatility in the market [10,11], resulting from high global demand and instability in supply. Between 1998 and 2018, the average price variation in soybean meal was approximately 36%. However, in certain periods of the year, its price can nearly double, increasing from about US$298/t to values close to US$570/t. This volatility is directly related to the concentration of global soybean production in three countries, Brazil, the United States, and Argentina, which together account for approximately 82% of global production [12,13]. Therefore, fluctuations in the supply and price of this ingredient directly affect feed costs, reducing profit margins and limiting expansion and technological investments in the poultry production chain.

In this context, peanut meal emerges as a still underutilized alternative [14]. This by-product of oil extraction is produced in more than 100 countries and generally presents a lower cost than soybean meal [15,16]. However, its use in poultry diets remains limited, mainly due to concerns regarding the presence of antinutritional factors, especially mycotoxins [17,18], as well as the presence of compounds such as phytate, tannins, lectins, and oligosaccharides [19]. In addition, previous studies on the use of peanut meal in poultry feeding have reported inconsistent performance results, making it difficult to establish clear recommendations for its inclusion in diets [2,3,4,20]. Given the scarcity of studies with laying hens, research with growing birds (broilers) has been used as a reference to evaluate the potential of peanut meal in poultry nutrition. Early findings indicate that the absence of amino acid supplementation may impair performance: El Boushy and Raterink [21] reported reduced body weight with a 10% inclusion level. In contrast, Costa et al. [4] showed that supplementation with lysine, methionine, threonine, and tryptophan allows inclusion levels of up to 20% without negative effects on performance; however, higher levels (32%) resulted in reduced body weight and poorer feed conversion, even with supplementation. Conversely, ref. [20] observed improvements in body weight and feed conversion with inclusion levels of up to 100%, combined with the addition of 5% fish meal, suggesting that different nutritional strategies may mitigate the limitations of this ingredient. In many cases, these inconsistencies are associated with the use of peanut meal without properly balancing the amino acid profile of the diet. However, this imbalance can be corrected through supplementation with industrial amino acids, which are currently widely available and economically accessible [14].

Furthermore, most available studies evaluate only zootechnical parameters, and there is a lack of research integrating productive performance, egg quality, economic viability of diets, and environmental impacts, such as greenhouse gas emissions [13,22]. Therefore, a knowledge gap remains regarding the potential of peanut meal as a substitute for soybean meal in egg production systems from a productive, economic, and environmental perspective. It is hypothesized that peanut meal can be included in laying hen diets without compromising productive performance or egg quality while potentially reducing feed costs and CO_2_ equivalent emissions. Given this context, this study was conducted to evaluate levels of peanut meal inclusion in diets for laying hens and their effects on productive performance, egg quality, economic viability of the diets, and CO_2_ equivalent emissions.

## 2. Materials and Methods

### 2.1. Ethical and Study Approvals

The trial was carried out at the Poultry Science Laboratory of the Department of Animal Science, UNESP, Jaboticabal Campus, São Paulo, Brazil. The Animal Ethics and Welfare Committee of São Paulo State University approved all experimental procedures used in this study under protocol number 9373488003/2025. The trial was conducted between September and November 2025 to evaluate the effects of peanut meal inclusion in diets for laying hens.

### 2.2. Housing, Experimental Design, and Management

A total of 200 Hisex White hens were housed in the experimental facilities, an open-sided shed equipped with a two-tier cage system, linear feeders, nipple drinkers, and six fans. The hens were selected based on body weight and egg production to obtain maximum uniformity when assembling the experimental units. Hens aged 72 weeks, with an average body weight of 1584 ± 80 g per bird and a mean egg production rate of 95% ± 2%, were selected and housed in metal cages measuring 0.5 × 0.45 × 0.45 m. The experimental design was completely randomized, with five treatments and ten replicates, totaling 50 experimental units. Each experimental unit consisted of four hens per cage. The experimental period lasted 70 days. Feed was provided daily at 110 g per hen, water was supplied ad libitum, and the lighting program consisted of 16 h of light and 8 h of darkness. Thermohygrometers were used to record temperature and humidity inside the house daily. During the trial, the average values for temperature were 21.0 °C, 27.5 °C, and 34.0 °C for minimum, mean, and maximum temperature, respectively, while the corresponding values for relative humidity were 34%, 53.9%, and 98% for minimum, mean, and maximum humidity.

### 2.3. Peanut Meal, Diets, and Treatments

Peanut meal was obtained from Sementes Esperança (Sementes Esperança Comércio Importação e Exportação Ltd.a., Jaboticabal, São Paulo, Brazil), which processes the product through mechanical pressing at 85 °C followed by chemical extraction with *n*-hexane. The guaranteed aflatoxin level in the peanut meal was 28 µg/kg. Based on this value, we present the calculated aflatoxin concentration in the diets, which ranged from 0 to 6.72 µg/kg and increased with increasing peanut meal inclusion.

The peanut meal used in this study was subjected to direct laboratory analysis to determine crude protein (Kjeldahl method—[23]), amino acid composition [24,25,26] and mineral composition for total mineral content (crude ash) or atomic absorption spectroscopy [27]. The values of apparent metabolizable energy corrected for nitrogen balance (ME_n_), amino acid profile, and mineral composition were determined in previous studies to adjust the diet formulations (Table S1). Amino acid digestibility coefficients were obtained from ref. [8].

The nutrient composition of the experimental diets was based on calculated values derived from ingredient composition, with corn and soybean meal previously analyzed by near-infrared spectroscopy (NIR) as part of routine quality control procedures prior to diet formulation. The experimental diets were formulated according to the feed composition tables and nutritional requirements described by ref. [8]. The treatments consisted of different inclusion levels of peanut meal in the diets of the birds as a replacement for soybean meal. Thus, Diet 1 (D1) to Diet 5 (D5), corresponding to treatments T1 to T5, corresponded to diets formulated with 0, 25, 50, 75, and 100% peanut meal, respectively (Table 1).

### 2.4. Experimental Procedures and Performance Variables

During the trial, the following variables were evaluated: feed intake (g/hen/day), egg production (%), egg weight (g), egg mass (g/day), and feed conversion ratio based on egg mass (kg/kg).

For feed intake, the feed provided to each replicate was weighed, and feed leftovers were weighed weekly to determine the weekly feed intake of each experimental unit.

To determine egg production, eggs were collected daily, and the number of eggs produced per replicate was recorded. To determine the average egg weight, eggs were weighed twice per week on consecutive days. Eggs were weighed by replicate and divided by the number of hens. Egg mass was calculated by multiplying the average egg weight by egg production. Finally, feed conversion ratio per egg mass was calculated as the ratio between feed intake per bird and egg mass [28].

### 2.5. Cumulative Effect on Egg Quality Variables

In the last week of the trial, three eggs were collected from each replicate on two alternate days, totaling six eggs per replicate and 300 eggs for the entire experiment. All eggs were identified, weighed using a semi-analytical balance, and immediately submitted for evaluation. The variables analyzed included average egg weight, absolute and relative weights of albumen, yolk, and shell, albumen height, Haugh unit, albumen diameter, albumen and yolk indices, yolk color, and eggshell thickness. Eggs were broken on a flat, smooth glass surface, where the height of the thick albumen and yolk was measured using a digital micrometer attached to a double-base stand.

Using albumen height and egg weight, the Haugh unit values were calculated [29]. The diameters of the thick albumen and yolk were measured using a digital caliper. The albumen and yolk indices were obtained by dividing their respective heights by the average values of their diameters. Egg components were weighed using an analytical balance with 0.01 g precision (Bel Engineering S1002, manufactured by Bel Engineering, Monza, Italy). After the eggs were broken, the shells were carefully washed with water and kept at room temperature for three days to dry before weighing. Eggshell thickness was measured with a caliper (0–150 mm; model 100-174BL Digimess, manufactured by Digimess Instrumentos, São Paulo, Brazil) [30].

### 2.6. Economic Variables of the Diets

To estimate costs, the prices of the ingredients included in the diets were considered (Table S2). Fixed costs: labor, equipment use, water, electricity, transportation, and facility maintenance were assumed to be constant across all treatments and were therefore not included in the feed cost. The average price of the ingredients was determined based on the mean market price over a 24-month period. The variable cost among treatments consisted of the inclusion level of peanut meal and other ingredients required to properly balance the diets (Table 1).

The economic variables analyzed were gross revenue (GR), calculated by multiplying the total number of eggs produced by the unit egg price of US$ 0.095 per egg, according to the following expression: GR (US$) = egg production × price. Feed cost (FC) was calculated as the product of the price per kilogram of diet and the total feed intake (tFI, kg), according to the expression: FC (US$) = tFI × diet price. Operating profit (OP, US$) was defined as gross revenue minus FC. The profitability index (PI, %) was defined as the percentage return after deducting FC, according to the expression: PI (%) = (OP ÷ GR) × 100. All monetary values were converted to U.S. dollars, and the formulas followed the definitions described by [31].

### 2.7. Carbon Footprint Assessment of Experimental Diets

The carbon footprint of the experimental diets was estimated using ingredient-specific greenhouse gas (GHG) emission factors expressed as kg CO_2_ equivalent (CO_2_ eq). These data were obtained from the Global Feed Life Cycle Assessment Institute (GFLI) database [32], which provides life cycle inventory information for feed ingredients and feed-related products. The emission factors used in the present study are presented in Tables S3 and S4.

The functional unit adopted was 1 ton of formulated feed. Therefore, the environmental assessment was conducted at the level of feed formulation, and the results represent the estimated GHG emissions associated with the production of the diets rather than the full environmental burden of egg production.

The system boundaries considered the upstream stages related to ingredient production and processing included in the diets: agricultural production of corn, industrial processing of soybean meal and peanut meal, extraction/production of soybean oil, and industrial production of synthetic amino acids. The assessment also included the carbon footprint associated with manure, using the digestibility coefficient of each diet multiplied by the feed intake of all hens of each treatment. According to the United States Environmental Protection Agency [33] the carbon footprint of manure is 4.2 kg CO_2_/kg. The carbon footprint of each diet was calculated by multiplying the inclusion level of each ingredient by its respective emission factor and summing the contributions of all ingredients in the formulation. Final results were expressed as kg CO_2_ eq. per ton of feed.

The same database source, emission factor framework, and calculation criteria were applied to all treatments to allow direct comparison among diets. Under this approach, differences in the carbon footprint estimates among treatments were interpreted as resulting from differences in ingredient composition, particularly the progressive replacement of soybean meal by peanut meal and the associated adjustments in supplemental amino acids and oil inclusion.

It is important to note that this analysis represents a partial and comparative carbon footprint assessment. It does not include ingredient transport to the feed mill, bird housing, electricity use, water use, egg collection, storage, packaging, or distribution. In addition, the study relied on secondary database values and did not generate primary life cycle inventory data for the specific ingredients used in the experiment. Thus, the estimates are subject to the assumptions, allocation procedures, and regional representativeness embedded in the GFLI database. For this reason, the environmental results should be interpreted as comparative estimates of the potential effect of dietary reformulation on carbon footprint and not as a complete life cycle assessment of the laying hen production system. This procedure has been applied in similar studies assessing environmental impacts in livestock production and agriculture [13,22].

### 2.8. Statistical Analysis

The experimental design was completely randomized, with five dietary treatments and ten replicates per treatment. The experimental unit was the cage containing four hens. For all variables, data from each experimental unit were summarized over the 70-day experimental period before analysis. Residuals were tested for normality and variances for homogeneity.

Data were analyzed by one-way ANOVA, considering dietary treatment as a fixed effect. When significant treatment effects were detected (*p* ≤ 0.05), means were compared with Dunnett’s test using the control treatment as the reference. In addition, polynomial regression analysis was performed to evaluate the linear and quadratic effects of increasing peanut meal inclusion levels. In this analysis, the peanut meal level was treated as a quantitative variable. Results are presented as means, standard error of the mean (SEM), and *p*-values. All statistical analyses were performed using SAS software (Statistical Analysis System, version 9.1), adopting 5% as the significance level.

## 3. Results

### 3.1. Productive Performance of Laying Hens

The results obtained for the productive performance variables of the birds are presented in Table 2. According to the ANOVA results, the probability values obtained were not significant (*p* > 0.05) for the treatment effect.

Therefore, the different inclusion levels of peanut meal did not affect the productive performance of the laying hens. The average daily feed intake was 100 g per hen (Table 2), demonstrating that dietary inclusion of peanut meal had no effect on feed intake. The average values for egg production, egg weight, egg mass, and feed conversion ratio were 94.8%, 60.5 g, 56.8 g/day, and 1.718 kg/kg, respectively. These results indicate that the inclusion of peanut meal did not affect the productive performance of the hens, regardless of the level of inclusion.

### 3.2. Internal and External Egg Quality

The results for egg quality are presented in Table 3. According to the ANOVA results, the probability values obtained were not significant (*p* > 0.05) for the treatment effect. Thus, the inclusion levels of peanut meal in the diets did not affect the weight of egg components: shell weight (5.7 g), yolk weight (16.6 g), and albumen weight (39.6 g), and consequently egg weight (60.5 g), evaluated in the last week as cumulative effect measurements. The qualitative variables indicating egg conformation, including albumen height, yolk height, albumen index, yolk index, and Haugh unit, were not affected by the inclusion levels of peanut meal in the diets. Likewise, the external egg quality measurements, shell percentage and shell thickness, remained unchanged and did not differ from the overall mean values (Table 3). These results indicate that the inclusion of peanut meal also did not affect egg quality, regardless of its level of inclusion in the diets.

### 3.3. Economic Analysis of the Diets

The results of the economic analysis are presented in Table 4. The variables used as inputs in the economic analysis, total feed intake and total number of eggs produced, did not show significant differences (*p* > 0.05), as expected, and are consistent with the productive performance results previously presented (Table 1). The economic variable gross revenue also did not differ significantly (*p* > 0.05), which was expected since it was calculated by multiplying the total number of eggs produced by a constant value, the egg price.

According to the ANOVA results, the probability values obtained for FC were significant (*p* < 0.05) for the treatment effect. Peanut meal inclusion levels of 25%, 50%, 75%, and 100% resulted in feed cost savings of US$ 4.80, US$ 16.77, US$ 29.22, and US$ 41.81 per ton of feed, respectively, compared with the reference diet (Table 4).

Linear regression analysis indicated that feed cost decreased linearly with increasing peanut meal inclusion level (FC = 1.74 − 0.055 × peanut meal level; R^2^ = 88%; *p* < 0.01; Table 5). The intercept represents the estimated feed cost for the control diet (0% peanut meal inclusion), whereas the negative slope indicates a progressive reduction in feed cost as soybean meal was replaced by peanut meal. Similarly, operating profit and income over feed cost increased linearly with increasing peanut meal inclusion. Linear regression models were also fitted for operating profit (OP = 4.56 + 0.054 × peanut meal level, R^2^ = 57%, Table 5) and profitability index (PI = 73.04 + 0.31 × peanut meal level, R^2^ = 86%, Table 5). The slopes of these models indicate that both OP and PI increased as peanut meal inclusion in the diets increased.

### 3.4. Footprint of the Diets

The five diets evaluated in the trial showed a reduction in environmental impact in terms of carbon footprint as the inclusion level of peanut meal increased in the diets. Compared with the reference diet, peanut meal inclusion levels of 25%, 50%, 75%, and 100% reduced emissions by 85.18, 185.57, 335.87 and 456.76 kg CO_2_ eq. per ton of feed, corresponding to percentage reductions of 4.0%, 8.9%, 15.8% and 21.5%, respectively, as shown in Table 4. The greatest reduction was observed at the 100% peanut meal inclusion level, avoiding the emission of 456.76 kg CO_2_ eq. per ton. These results indicate that peanut meal may be an alternative for more sustainable production by significantly reducing CO_2_ equivalent emissions (Table 4).

## 4. Discussion

This study evaluated the inclusion of peanut meal in diets for commercial laying hens at 72 weeks of age. The results obtained after 10 weeks indicated favorable effects of peanut meal inclusion on feed-related economic variables and a lower estimated carbon footprint of the diets, suggesting that peanut meal may be a promising alternative ingredient in laying hen feed formulation.

The results of this study complement the findings of ref. [2], who, after six weeks of experimentation, also indicated that peanut meal levels did not affect productive performance or egg quality. These authors suggested that an economic analysis would probably indicate peanut meal as a viable option, and, in the present study, the results demonstrate that the hypothesis of economic feasibility of using peanut meal is acceptable. In this study, correction of the amino acid profile was applied by supplementing L-valine and L-isoleucine, in addition to L-methionine, L-lysine, L-threonine, and L-tryptophan used by [2] (Table 1). This result is important and goes beyond the economic analysis because it demonstrates that the use of industrial amino acids (in this case, all the main amino acids commercially available) allowed precise correction of the birds’ requirements, ensured performance, and maintained the effect of peanut meal on feed cost.

Unlike ref. [2], no mycotoxin adsorbent or phytase was included in the present study, and the experimental period was longer. Although no statistically detectable adverse effects were observed on feed intake, egg production, or egg weight during the experimental period, these findings should not be interpreted as evidence of the absence of mycotoxin contamination, since no analytical determination of aflatoxins was performed in the diet batches.

Intentionally, a period longer than 21 days was used, which has been described as the minimum time required for birds to show the first symptoms derived from contamination by mycotoxins when present in low concentrations in feed. Previous studies demonstrated that 56 days of experimentation [34] were sufficient for birds to present symptoms affecting feed consumption. Therefore, the absence of effects on feed intake and other variables, such as egg production and egg weight, both related to feed intake, supports the hypothesis that there was no adverse effect associated with mycotoxins originating from the inclusion of peanut meal, especially at the highest inclusion levels of 120, 180, and 240 kg of peanut meal per ton of feed. However, because aflatoxins were not analytically determined in diet batches, these findings cannot be interpreted as evidence of the absence of mycotoxin contamination, although they may suggest a certain degree of tolerance of the birds to the possible presence of mycotoxins at the inclusion levels evaluated. These correspond approximately to the replacement of 50%, 75%, and 100% of soybean meal in the birds’ diet.

Although no treatment effect (*p* > 0.05) was detected for egg production (Table 2), an analysis of the genetic potential of the birds during the experimental period is warranted. Despite the birds being 72 weeks old, the overall mean egg production was 94.8%, a level close to peak production, which is precisely 2% below the maximum potential described by the genetic line. Therefore, the results obtained in this study may be applied to situations in which the physiological stage or productive potential of the bird is similar to that described, extrapolating chronological age. Although the recommendation for replacing soybean meal with peanut meal was similar to the findings of [2], the genetic egg production potential of Rugao line hens at 28 weeks of age was 71.2%, which is below the potential described in the present study. This reinforces that the physiological stage should be considered rather than chronological age.

A convergence between the results obtained in this study and previous studies by [2] and ref. [3] is the better response observed for diets based on corn and soybean meal. These studies show a difference that, although not statistically significant, may have practical importance, particularly in guiding future research for better use of feed resources. In general, egg production was approximately one percentage point (1%) higher; however, under a scenario of unchanged feed intake, feed conversion inevitably increased. In this study, feed intake increased by approximately 40 g to produce 1 kg of egg mass, which corresponds to 110 kcal of energy (2770 kcal/kg × 0.04 kg) at the maximum inclusion level of peanut meal. As mentioned, no opposite result favoring peanut meal inclusion was observed. Therefore, a hypothesis arises that the use of enzymes, especially carbohydrases, may assist in the dynamics of the digestive process, since phytase was used by [2] while the scenario described here remained unchanged.

The amino acid profile of peanut meal differs from that of soybean meal, and the simplest way to characterize this difference is by the concentration of essential amino acids (EAA) and non-essential amino acids (NEAA). In peanut meal, the proportion is approximately 36%:64%, whereas in soybean meal it is 45%:55% (EAA:NEAA), respectively. This corresponds to a difference of approximately 20% (EAA 36% ÷ EAA 45%). Arginine is the only essential amino acid present in higher concentration in peanut meal compared with soybean meal, while the others (lysine −58%, methionine −49%, threonine −34%, tryptophan −19%, arginine +36%, valine −27%, isoleucine −37%, leucine −21%, histidine −17%, phenylalanine −17%) occur at lower concentrations in peanut meal protein than in soybean meal protein, especially lysine, methionine, threonine, and isoleucine.

Therefore, nutritional strategies such as phytase, carbohydrases, and mycotoxin adsorbents, although very useful for optimizing the use of feed resources, cannot replace the need to consider differences in the essential amino acid profile during diet formulation in order to ensure the maintenance of bird performance. The literature includes several reports [4,17,20,35] in which the use of peanut meal impaired bird performance. These studies commonly, when they considered amino acid supplementation, focused on the main or most commonly used amino acids such as methionine, lysine, and threonine, rather than correcting the entire amino acid profile required by the birds.

The results for egg weight, yolk percentage, albumen percentage, albumen height, and Haugh unit observed in this study (Table 3) are consistent with those reported by [36], who evaluated these variables in different laying hen strains (Babcock B300, Hy-Line W36, Lohmann White, and Hisex) at 69 weeks of age. Ref. [2] did not observe any effect of peanut meal inclusion levels on egg quality variables (Haugh unit, albumen percentage, yolk percentage, yolk color, shell percentage, and shell thickness) in Rugao laying hens at 28 weeks of age. These results are similar to those observed in the present study and reinforce that diets formulated with peanut meal, when properly balanced, do not interfere with egg quality [37].

The yolk and albumen index results obtained in the present trial fall within the range considered excellent for egg quality in all treatments, as reported in several studies [38,39]. These yolk index values demonstrate the proper balance between yolk and albumen proportions. A lower yolk index indicates migration of water from the albumen to the yolk, reducing its volume as a consequence of increased permeability of the vitelline membrane [40]. Eggs with yolk index values below the standard indicate low resistance of the vitelline membrane, making yolks more susceptible to rupture during egg handling and consequently resulting in lower egg quality [41]. This result demonstrates that peanut meal, regardless of its inclusion level, does not affect this parameter of internal egg quality (Table 3).

Ref. [40] determined that the shell thickness of eggs of excellent quality ranges from 300 to 400 µm, which helps prevent the loss of internal egg quality through evaporation of internal contents such as carbon dioxide and water, thereby maintaining the integrity of internal egg structures. The results of the present study (Table 3) are consistent with these shell thickness parameters and are similar to those obtained by [42,43,44]. According to [40], 9–12% of egg weight corresponds to the eggshell. These values are consistent with those observed in the present study, demonstrating that diets formulated with peanut meal did not affect shell thickness, which could compromise the integrity of the shell structure, albumen, or yolk. According to ref. [45], among several parameters that determine eggshell resistance, shell thickness is the most important. Similar results for shell weight and thickness to those obtained in the present study were observed by [46] in research with 24-week-old Isa Brown laying hens evaluating the effect of egg weight on internal and external egg quality.

Haugh units are widely used as an indicator of egg quality. Several studies [36,40,47] report that Haugh unit values above 72 indicate eggs of excellent quality in terms of freshness, a result observed in all treatments of the present study (Table 3). Ref. [3], investigating the use of peanut meal in diets for laying hens, reported that Haugh unit values did not differ significantly compared with birds fed diets based entirely on soybean meal. These findings corroborate the results obtained in the present experiment.

The albumen and yolk percentages of high-quality eggs generally range between 56–63% and 24–30%, respectively [40]. In the present study, the albumen and yolk indices were within the range established as excellent (Table 3). Albumen and yolk heights were consistent with those reported by [48], who studied the effect of egg weight on egg quality characteristics in 33-week-old Lohmann laying hens. Eggs with weights similar to those observed in the present experiment showed similar albumen and yolk heights. These results are also consistent with those reported by [29], who investigated the relationship between albumen measurements and the functional properties of albumen in Brown Leghorn laying hens.

With regard to the economic analysis, the inclusion level of 100% peanut meal resulted in the lowest feeding costs, with a reduction of US$ 0.23 per kg of feed compared with the corn–soybean meal diet (Table 4). The regression analysis of feed cost showed a decreasing linear equation, which allowed a more reliable recommendation for the inclusion level of 100% peanut meal (Table 5). In the present study, the observed feed cost saving per kilogram was US$0.04 at the inclusion level of 100% peanut meal replacing soybean meal (Table 4). This indicates that peanut meal provides better economic efficiency in diet formulation.

Ref. [31] reported a reduction in the cost of laying hen diets when using 5% tomato residue meal as a protein source, partially replacing soybean meal. This resulted in savings of R$ 26.00 per ton of feed compared with the reference diet, without compromising the zootechnical performance of the birds. Unlike tomato residue meal, peanut meal can fully replace soybean meal, which further increases the reduction in dietary costs. An economic analysis of the inclusion of 20% tucumã residue meal in diets for commercial laying hens showed a saving of R$ 0.40 per kilogram in feed price [49,50]. These results demonstrate that alternative feed ingredients can reduce poultry nutrition costs, which account for about 70% of the total production costs, especially when the alternative ingredient replaces the main protein source (soybean meal) or the main energy source (corn), both of which constitute the largest proportions of poultry diets.

Regarding environmental impact, specifically the carbon footprint component, ref. [51] recommends that such studies include the economic component in addition to the environmental one, since otherwise an environmentally favorable solution could inadvertently prove economically unsustainable. In the present study, both components were considered, and peanut meal showed better results than soybean meal in both aspects (Table 4). The use of peanut meal is not only cheaper than soybean meal but also more sustainable in comparison. A study by ref. [52], which evaluated the environmental impact of using corn gluten as a protein source partially replacing soybean meal and including proteases, showed a reduced environmental impact of alternative diets compared with the traditional soybean meal diet. These results support the idea that replacing ingredients with alternatives can have direct impacts on environmental sustainability and can significantly mitigate GHG emissions (Table 4). Ref. [53], in research on alternative ingredients replacing soybean meal and corn in broiler and laying hen diets in Thailand, reported reductions of 39.6% and 49.6% in emissions of kg CO_2_ eq/ton of feed, respectively.

Another study [54] evaluating rye as an alternative ingredient to corn, with inclusion levels of 4–25% in broiler diets aimed at reducing CO_2_ emissions, showed reductions in emissions of kg CO_2_ eq/ton of feed ranging from 3.1% to 7.8%. Ref. [55], investigating the economic value of the environmental impact of soybean meal in poultry and swine diets with crude protein levels ranging from 44% to 48%, observed in poultry diets that formulations using soybean meal with 48% crude protein reduced emissions of kg CO_2_ eq/kg by 20.4% compared with soybean meal containing 44% crude protein. Therefore, this reduction in emissions did not increase feed costs. The studies presented above demonstrate that the use of alternative ingredients with lower CO_2_ emissions can reduce emissions of kg CO_2_ eq/ton of feed, contributing to environmentally sustainable poultry production and providing a database for diet formulations that better respond to current environmental challenges.

## 5. Conclusions

Under the conditions of the present study, increasing the dietary inclusion of peanut meal up to the complete replacement of soybean meal did not negatively affect productive performance or egg quality variables in laying hens from 72 to 80 weeks of age. In addition, peanut meal inclusion reduced feed cost and lowered the estimated carbon footprint of the diets at the formulation level. Therefore, peanut meal may represent a promising alternative protein ingredient for laying hens under similar nutritional and management conditions. However, these findings should be interpreted within the limitations of the experimental conditions and the partial nature of the environmental assessment.

## Figures and Tables

**Table 1:** Composition of experimental diets.

Ingredients	Control	Levels of Peanut Meal in the Diets			
	Diet	D2	D3	D4	D5
	D1	60 kg/ton	120 kg/ton	180 kg/ton	240 kg/ton
Corn	648.235	630.238	629.6	629.6	629.6
Soybean meal 46	237.244	191.13	129.28	65.37	1.723
Soybean oil	1.195	4.327	4.482	4.928	5.142
Peanut meal 45	0	60	120	180	240
Dicalcium phosphate	10.857	10.78	10.836	10.915	10.991
Limestone	93.796	93.966	94.185	94.409	94.632
NaCl	3.999	3.952	3.907	3.864	3.82
L-Methionine 100	1.974	2.154	2.467	2.803	3.135
L-Lysine HCl 79		0.753	1.982	3.278	4.565
L-Threonine PRO 80			0.092	0.61	1.124
L-Valine 96.5			0.342	0.724	1.101
L-Isoleucine 90			0.127	0.703	1.274
L-Tryptophan 98				0.097	0.193
Choline chloride 60	1.2	1.2	1.2	1.2	1.2
Mineral premix ^1^	0.5	0.5	0.5	0.5	0.5
Vitamin premix ^2^	1.0	1.0	1.0	1.0	1.0
Calculated nutrients values					
Metabolizable energy (kcal/kg)	2770	2770	2770	2775	2778
Crude fiber (%)	2.71	2.83	2.88	2.93	2.98
Fat (%)	2.73	2.91	2.81	2.73	2.62
Linoleic acid (%)	1.51	1.61	1.58	1.56	1.53
Total choline (%)	1563	1531	1469	1402	1336
Crude protein (%)	16.22	16.74	16.76	16.77	16.79
Lysine (%)	0.740	0.740	0.740	0.740	0.740
Methionine (%)	0.428	0.434	0.447	0.461	0.474
Methionine + Cysteine (%)	0.659	0.659	0.659	0.659	0.659
Threonine (%)	0.560	0.542	0.511	0.511	0.511
Tryptophan (%)	0.179	0.179	0.170	0.170	0.170
Arginine (%)	0.975	1.091	1.163	1.229	1.295
Valine (%)	0.673	0.666	0.666	0.666	0.666
Isoleucine (%)	0.613	0.592	0.555	0.555	0.555
Leucine (%)	1.349	1.340	1.296	1.246	1.197
Potassium (%)	0.642	0.626	0.588	0.546	0.505
Sodium (%)	0.170	0.170	0.170	0.170	0.170
Total calcium (%)	3.90	3.90	3.90	3.90	3.90
Available phosphorus (%)	0.29	0.29	0.29	0.29	0.29

^1^ Mineral premix: Iron (min) 100 g, Copper (min) 20 g, Manganese (min) 130 g, Zinc (min) 130 g, Iodine (min) 2000 mg. ^2^ Vitamin premix: Vitamin A (min) 11,000,000 IU, Vitamin D_3_ (min) 4,000,000 IU, Vitamin E (min) 55,000 IU, Vitamin K_3_ (min) 3000 mg, Vitamin B_1_ (min) 2300 mg, Vitamin B_2_ (min) 7000 mg, Pantothenic acid (min) 12 g, Vitamin B_6_ (min) 4000 mg, Vitamin B_12_ (min) 25,000 mcg, Nicotinic acid (min) 60 g, Folic acid (min) 2000 mg, Biotin (min) 250 mg, Selenium (min) 300 mg.

**Table 2:** Means, standard error of the mean (SEM), and probability values for feed intake, egg production, egg weight, egg mass, and feed conversion ratio of laying hens from 72 to 80 weeks of age fed diets containing levels of peanut meal.

Treatments	Level (%)	Feed Intake	Egg Production	Egg Weight	Egg Mass	Feed Conversion Ratio
Diet 1	0	100.2	96.1	60.4	58.0	1.701
Diet 2	25	99.9	94.0	61.7	56.6	1.712
Diet 3	50	99.9	94.4	60.7	56.3	1.700
Diet 4	75	100.5	94.4	60.0	57.5	1.738
Diet 5	100	99.4	95.0	59.9	56.9	1.740
General		100.0	94.8	60.5	56.8	1.718
SEM		0.064	0.116	0.102	0.097	0.003
*p*-value F ANOVA		>0.05	>0.05	>0.05	>0.05	>0.05
CV ^1^, %		4.03	3.92	3.13	4.68	5.4

^1^ Coefficient variation.

**Table 3:** Means, standard error of the mean (SEM), and probability values for egg weight (g), shell weight (g), yolk weight (g), albumen weight (g), yolk color, albumen height (mm), yolk height (mm), shell thickness (µm), shell percentage (%), yolk percentage (%), albumen percentage (%), albumen index (%), yolk index (%), and Haugh unit (%) of laying hens from 72 to 80 weeks of age fed diets containing different levels of peanut meal.

Variables	Levels of Peanut Meal in Diet					General	SEM	*p*-Value F ANOVA	CV ^1^ %
	0%	25%	50%	75%	100%				
Egg weight	60.4	61.7	60.7	60.0	59.9	60.5	0.09	0.501	3.13
Eggshell weight	5.7	5.6	5.7	5.6	5.7	5.7	0.03	0.560	2.95
Yolk weight	16.8	16.8	16.4	16.8	16.4	16.6	0.11	0.480	4.75
Albumen weight	40.3	40.6	39.3	38.4	39.4	39.6	0.34	0.190	5.58
Yolk color	5.6	5.7	5.6	5.5	5.7	5.6	0.04	0.453	5.09
Albumen height	8.3	8.4	8.4	8.1	8.7	8.4	0.12	0.590	10.44
Yolk height	17.2	17.4	17.3	17.4	17.4	17.4	0.09	0.970	3.62
Eggshell thickness	398.0	373.0	374.0	366.0	376.0	373.0	5.00	0.940	9.97
Shell	9.1	9.3	9.3	9.1	9.2	9.2	0.07	0.770	5.11
Yolk	26.8	26.6	26.7	27.7	27.5	27.1	0.16	0.069	3.87
Albumen	64.1	64.2	64.0	63.2	63.3	63.8	0.19	0.321	2.09
Albumen index	0.112	0.114	0.116	0.112	0.123	0.110	0.001	0.327	11.56
Yolk index	0.422	0.425	0.425	0.424	0.429	0.425	0.02	0.910	3.66
Haugh unit	89.9	90.6	91.3	89.2	92.8	90.8	0.64	0.478	5.02

^1^ Coefficient variation.

**Table 4:** Means, standard error of the mean (SEM), and probability values for price of feed, footprint of the diets, total feed intake (tFI, kg), total egg production (tEP, unity), revenue balance (RB, US$), feed cost (FC, US$), operational profit (OP, US$), and income over feed cost (IP, %) of laying hens from 72 to 80 weeks of age fed diets containing different levels of peanut meal.

Treatments	Level (%)	Price of Feed	Footprint of the Diets	tFI	tEP	RB (U$)	FC	OP	IP
Diet 1	0	0.23	2124.4	6.9	66.1	6.3	1.7 a	4.6 b	73.6 d
Diet 2	25	0.22	2039.2	6.8	64.9	6.2	1.6 b	4.7 b	74.0 d
Diet 3	50	0.21	1935.8	6.8	65.1	6.2	1.5 c	4.7 b	75.3 c
Diet 4	75	0.20	1788.5	6.9	66.0	6.2	1.5 c	4.8 a	76.4 b
Diet 5	100	0.19	1667.6	6.8	65.8	6.2	1.4 d	4.9 a	77.8 a
General				6.8	65.6	6.2	1.5	4.7	75.5
SEM				0.03	0.19	0.02	0.02	0.03	0.32
*p*-value F ANOVA				>0.05	>0.05	>0.05	0.0001	0.0016	0.0001
Coefficient Variation, %				2.62	1.93	1.32	2.87	2.08	0.93

a–d: Different lowercase letters within the same column indicate significant differences by Dunnett’s test (*p* < 0.05).

**Table 5:** Estimates of the intercept and slope values of linear models for feed cost (US$), operational profit (US$), and income over feed cost (%), coefficient of determination, and significance of the fitted models for laying hens from 72 to 80 weeks of age fed diets containing different levels of peanut meal.

Variables	Intercept	Slope	R^2^	Significance
Feed cost (US$)	1.74	−0.055	87.67	<0.01
Operational profit (US$)	4.56	0.054	56.57	<0.01
Income over feed cost (%)	73.04	0.31	85.56	<0.01

R^2^: Coefficient of determination.

## Data Availability

The data presented in this study are available on request from the corresponding author.

## Supplementary Materials

The following supporting information can be downloaded at: https://www.mdpi.com/article/10.3390/ani16101541/s1. Table S1: Peanut meal composition; Table S2: Average prices of the ingredients used in diet formulation over the last two years; Table S3: Carbon dioxide equivalent (CO_2_ eq.) emissions of ingredients from farm production to processing ^1^; Table S4: Environmental impact potential associated with the production and delivery of 1 kg of each ingredient or amino acid ^1^.

## Abbreviations

The following abbreviations are used in this manuscript:

ANOVA	Analysis of Variance
CO_2_ eq	Carbon dioxide equivalent
CV	Coefficient of Variation
EAA	Essential Amino Acids
FC	Feed Cost
GHG	Greenhouse Gas
GFLI	Global Feed Life Cycle Assessment Institute
GR	Gross Revenue
IP	Income over Feed Cost
LCA	Life Cycle Assessment
MEn	Apparent Metabolizable Energy corrected for nitrogen balance
NEAA	Non-Essential Amino Acids
OP	Operating Profit
SEM	Standard Error of the Mean
tFI	Total Feed Intake
tEP	Total Egg Production
