# Supplementary material for: Peanut Meal as a Sustainable Alternative to Soybean Meal in Laying Hen Diets
Source: Animals (Basel). 2026 May 18;16(10):1541. doi: 10.3390/ani16101541 (PMC13203734; doi:10.3390/ani16101541)
Supplement: Supplementary file 1 [file animals-16-01541-s001.zip › animals-4246228-supplementary.pdf]

Supplementary Materials:

Tables S1. Peanut meal composition.

**Constituent**	**Unity**	**Value**
Dry matter[1]	%	89.8
Crude protein[1]	%	45.00
Crude fiber[1]	%	6.88
Fat[1]	%	0.91
Metabolizable energy[2]	kcal/kg	2324.0
Lysine[1]	%	1.06
Methionine[1]	%	0.29
Methionine + Cystine[1]	%	0.68
Threonine[1]	%	0.98
Tryptophan[1]	%	0.47
Arginine[1]	%	4.48
Glycine + Serine[1]	%	4.43
Valine[1]	%	1.50
Isoleucine[1]	%	1.19
Leucine[1]	%	2.58
Histidine[1]	%	0.90
Phenylalanine[1]		1.96
Phenylalanine + Tyrosine[1]	%	3.63
Ash[1]	%	5.82
Potassium[2]	%	1.25
Sodium[1]	%	0.05
Chloride[2]	%	0.05
Calcium[1]	%	0.20
Total phosphorus[1]		0.63
Phytic P[2]		0.42
Available P[2]	%	0.21
Choline[3]	mg/kg	1655
Linoleic acid[2]	%	0.24
Aflatoxin[1]	µg/kg	28

[1]Analyzed; [2] Ref. [8]; [3]Ref. [9].

Table S2. Average prices of the ingredients used in diet formulation over the last two years.

Ingredients	Price US$	Unit	Fonte
Corn	190,930	ton	USA[1]
Soybean meal 46%	454,590	ton	USA[1]
Soybean oil	1121,550	ton	USA[1]
Peanut meal 45%	268,080	ton	Location[1]
Dicalcium phosphate	4,990	kg	Location[1]
Limestone	17,54	ton	Location[1]
Sodium chloride	30,0	Ton	Location[1]
L-methionine 100%	2,530	kg	Location[1]
L-lysine HCl 79%	1,277	kg	Location[1]
L-threonine 80%	1,347	kg	Location[1]
L-tryptophan 98%	8,319	kg	Location[1]
L-valine 96.5%	2,435	kg	Location[1]
L-isoleucine 90%	11,000	kg	Location[1]
Mineral premix	1,80	kg	Location[1]
Vitamin premix	3,90	kg	Location[1]

[1]United States of America; [2]Jaboticbal, City, São Paulo - Brazil.

Table S3. Carbon dioxide equivalent (CO_2 eq.) emissions of ingredients from farm production to processing[1].

Ingredients	kg CO_2 eq./ton	Level
Corn production (BRA)	895.7	Farm - level
Soybean production (BRA)	1,873.2	Farm - level
Soybean meal (BRA)	1,576.7	Feed - level
Soybean oil (BRA)	3,965.7	Feed - level
Peanut production (USA)	960.5	Feed - level
Peanut meal (USA)	289.2	Feed - level
Corn processing (USA)	376.0	Feed - level

[1]Source: GFLI; Global Feed Life Cycle Assessment Institute (GFLI) database;
BRA, Brazil;
USA, United States of America;
Carbon dioxide emission of manure: 4.2 CO_2 eq/kg (Environmental protection agency, 2015; Emission factors of greenhouse gas inventories. https://www.epa.gov/sites/production/files/2015-07/documents/emission-factors_2014.pdf

Table S4. Environmental impact potential associated with the production and delivery of 1 kg of each ingredient or amino acid[1].

Ingredients	kg CO_2 eq. per kg of ingredient produced
L-lysine	4.300
L-threonine	4.300
L-methionine	3.050
L-tryptophan	8.540
L-valine	8.540
L-isoleucine[2]	7.270

[1] Ref. [56]
[2]Amino acids | Verified by CarbonCloud
